# Several One-Domain Zinc Finger µ-Proteins of Haloferax Volcanii Are Important for Stress Adaptation, Biofilm Formation, and Swarming

**DOI:** 10.3390/genes10050361

**Published:** 2019-05-10

**Authors:** Chantal Nagel, Anja Machulla, Sebastian Zahn, Jörg Soppa

**Affiliations:** Department of Biosciences, Institute for Molecular Biosciences, Goethe-University, Max-von-Laue-Str. 9, D-60438 Frankfurt, Germany; ChantalNagel@web.de (C.N.); Anja.Machulla@gmx.de (A.M.); Zahn@bio.uni-frankfurt.de (S.Z.)

**Keywords:** Archaea, *Haloferax volcanii*, zinc finger protein, small protein, µ-protein, deletion mutant, biofilm, swarming, phenotypic analysis

## Abstract

Zinc finger domains are highly structured and can mediate interactions to DNA, RNA, proteins, lipids, and small molecules. Accordingly, zinc finger proteins are very versatile and involved in many biological functions. Eukaryotes contain a wealth of zinc finger proteins, but zinc finger proteins have also been found in archaea and bacteria. Large zinc finger proteins have been well studied, however, in stark contrast, single domain zinc finger µ-proteins of less than 70 amino acids have not been studied at all, with one single exception. Therefore, 16 zinc finger µ-proteins of the haloarchaeon *Haloferax volcanii* were chosen and in frame deletion mutants of the cognate genes were generated. The phenotypes of mutants and wild-type were compared under eight different conditions, which were chosen to represent various pathways and involve many genes. None of the mutants differed from the wild-type under optimal or near-optimal conditions. However, 12 of the 16 mutants exhibited a phenotypic difference under at least one of the four following conditions: Growth in synthetic medium with glycerol, growth in the presence of bile acids, biofilm formation, and swarming. In total, 16 loss of function and 11 gain of function phenotypes were observed. Five mutants indicated counter-regulation of a sessile versus a motile life style in *H. volcanii*. In conclusion, the generation and analysis of a set of deletion mutants demonstrated the high importance of zinc finger µ-proteins for various biological functions, and it will be the basis for future mechanistic insight.

## 1. Introduction

For a long time, very small proteins (µ-proteins) have been nearly totally neglected. Genome annotations typically used a lower limit of 100 codons to include an open reading frame (ORF) as a predicted protein-coding gene. On the one hand, the annotation of a vast number of false-positive genes was prevented, on the other hand, real genes for µ-proteins were also not included and thus they escaped attention. In addition, experimental protein-analytical methods had been previously optimized for normal-sized proteins, which led to the loss of µ-proteins during purification or analyses.

During the last years the awareness has emerged that many µ-proteins do exist in prokaryotes, as well as in eukaryotes, and that they have important biological functions. Several recent reviews summarize the current knowledge about µ-proteins in prokaryotes [1,2,3] and in eukaryotes [4,5,6]. For higher eukaryotes, two databases for µ-proteins have been established during the last two years [7,8]. For humans, the database SmProt contains more than 100,000 small proteins of less than 100 aa (http://bioinfo.ibp.ac.cn/SmProt). The field is so new, that generally accepted definitions and a generally accepted terminology do not yet exist. Upper limits of 50 amino acids (aa), 70 aa, or 100 aa are used to define the group of very small proteins. The terms “small proteins”, “peptides”, “microproteins”, “micropeptides”, and “sORFs-encoded peptides” are in use. In this contribution we will use an upper limit of 70 aa and the term “µ-proteins”, to distinguish between “small proteins”, which could be understood as proteins smaller than an average protein of about 300 aa, and also between “peptides”, which could include very small peptides of 10 to 30 aa, which are not encoded by distinct ORFs.

The emerging field of µ-protein research is mainly driven by improvements in two experimental approaches. Ribosomal profiling allows the highly parallel determination of the positions of all ribosomes of a cell on all transcripts [4,9]. All detected transcripts are proven to be translated into proteins, including small transcripts that encode µ-proteins. Another approach is the detection of µ-proteins by mass spectrometry (MS). Because standard proteomic approaches failed to detect µ-proteins, different steps had to be optimized, and the optimized procedure for small proteins is often called peptidomics [10]. The Oesterhelt group used optimized procedures to characterize the low molecular weight proteome of the halophilic archaeon *Halobacterium salinarum* already more than ten years ago [11]. Three hundred and eighty small proteins could be experimentally verified. The majority of these proteins (62%) had no assigned function, underscoring that the small proteome is understudied also in haloarchaea. It was noted that the group of small proteins with unknown function included 20 proteins that contained two C(P)XCG motifs. It was predicted that the four cysteines might complex a zinc ion and the proteins might thus contain zinc fingers [11].

Zinc finger proteins were first discovered in eukaryotes and were long thought to be confined to this domain [12]. They are abundant in eukaryotes and are involved in numerous processes, e.g., regulation of transcription, protein degradation, signal transduction, and many others [13]. Zinc finger domains are interaction modules that can enable the binding of zinc finger proteins to DNA, RNA, other proteins, lipids, and small molecules [14,15]. Therefore, the identification of zinc finger motifs is only indicative for zinc binding, but does not allow to predict protein function or association partners. The “classical” and most studied zinc finger domain contains two cysteines and two histidines (C2H2 zinc fingers). However, C3H and C4 zinc finger domains also exist. The four amino acids that coordinate the zinc ion are found in two motifs. The classical C2H2 zinc finger contains the motifs CXXC and HXXH (X = any amino acid). Many C4 zinc fingers contain two C(P)XCG motifs (P in brackets, because only one of the two motifs contain a proline). However, also many variants of these motifs exist, e.g., with slight differences in the distance of the two cysteines or histidines. Accordingly, zinc finger domains exhibit high structural variability, and they have been divided into eight structural groups [16]. A zinc finger is often used as a generic term for all subclasses, which, however, also have specific names like zinc knuckle, zinc ribbon, or treble clef [16].

Zinc finger proteins have also been found in bacteria, the first one was the Ros protein from *Agrobacterium tumefaciens* [17]. Ros is a transcription factor that regulates the *ipt* gene encoding isopentenyl transferase. Ros binds to an inverted repeat in the *ipt* promoter and represses transcription. Ros contains a classical C2H2 zinc finger, and homologues have been identified in additional bacteria and in archaea [18,19]. In addition, also a few examples of C4 zinc finger proteins have been found in bacteria and in archaea [20,21].

Most zinc finger motifs are parts of larger proteins, and the number of small zinc finger proteins is rather low. A bioinformatics search in archaeal and bacterial genomes with very relaxed patterns (e.g., CXXCX, HXXCX, HXXHX, etc.) revealed that only 1.5% of bacterial small proteins of less than 100 aa are potential zinc finger proteins, while, in contrast, this is true for 8% of archaeal small proteins [22]. The functions of very few small zinc finger proteins are known, e.g., they are small subunits of the RNA polymerase or the ribosome. However, most of them do not have an annotated function.

One of experimentally identified 20 C(P)XCG proteins of *H. salinarum* (see above) [11] was studied in detail. It was shown that it was a positive regulator of its adjacent gene, *bop* (bacterioopsin), and of *crtB1* (phytoene synthase) [22]. The expression of both genes was severely diminished in mutants of this zinc finger gene, which was named *brz* (bacterioopsin-regulating zinc finger protein). To our knowledge, this is the only experimental study of a small putative zinc finger protein in all three domains of life, which is not a subunit of a larger protein complex. Therefore, we decided to study more systematically whether small zinc finger proteins have important biological functions. We used the haloarchaeal model species *Haloferax volcanii*, because genetic (and other) tools are highly developed [23], and, very recently it was nominated as the “Microbe of the month” [24]. Its genome annotation is of very high quality, because it is constantly manually curated and many other haloarchaeal genomes are available for comparisons [25]. Sixteen genes for putative zinc-finger µ-proteins were chosen, and in frame deletion mutants were generated. The phenotypes of the mutants were compared to that of the wild-type under eight different conditions, which were chosen to test many different genes and pathways. For 12 of the mutants, phenotypic differences could be observed under at least one condition, revealing that many of these proteins fulfill important functions in *H. volcanii.*

## 2. Materials and Methods

### 2.1. Strains and Media

The *H. volcanii* strain H26 [26] was used in this study as a wild-type for construction of the 16 deletion mutants. It contains a deletion in the *pyrE2* gene, which enables two selection steps during mutant construction, and, thereby, facilitates and accelerates the procedure. It was grown in a complex medium with 2.1 M NaCl at 42 °C and good aeration (220 rpm) [27].

The *Escherichia coli* strain XL1-blue MRF’ (Agilent Technologies, Waldbronn, Germany) was used for cloning and was grown in standard media [28].

### 2.2. Generation of in Frame Deletion Mutants

The 16 in frame deletion mutants were generated by the so called Pop-In-Pop-Out method as described previously [26,29]. In short, for each deletion strain two PCR fragments of about 500 bpwere generated that contained (1) the upstream region and the first codons of the respective gene, and (2) the last codons and the downstream region. The two PCR fragments had an overlap, so that they could be fused by a third PCR reaction, and the resulting PCR fragment was inserted into the plasmid pMH101 by restriction selection cloning [30]. All oligonucleotide sequences are summarized in Supplementary Table S1. The sequences of the 16 plasmids were verified by sequencing. The plasmids were used to transform *H. volcanii* H26, and the Pop-In clones, which had integrated the plasmid into the genome, were selected by growth in the absence of uracil (the plasmids contain the *pyrE2* gene, which had been deleted from the genome of H26). Subsequently, the Pop-In variants were grown in medium with uracil and 5′-FOA, which selects for the absence of the *pyrE2* gene and thus for the Pop-Out variants. The initial identification of Pop-In clones and Pop-Out clones was performed by colony PCR. *H. volcanii* is polyploid [31], therefore, great care has to be taken that the Pop-Out clones are homozygous and do not contain one or a few remaining copies of the wild-type genome. Therefore, genomic DNA was isolated from candidate clones, and PCR with a large number of cycles, as well as Southern blot analyses, were used for the identification of homozygous deletion mutants. The deleted genomic regions of the 16 in frame deletion mutants are listed in Supplementary Table S2.

### 2.3. Growth Analyses

*H. volcanii* can be grown in microtiter plates, which enables parallel growth of many cultures and greatly facilitates phenotypic analyses of mutant collections under different conditions [32]. The complex medium, as well as a synthetic medium with different carbon sources, were used [27], as described in the text. Genomic DNA as phosphate source was added at a final concentration of 250 µg/mL, as described [33]. For each condition, 150 µL medium was inoculated to an OD_600_ of 0.05 from a preculture that had been grown under the respective condition. The cultures were grown on a Heidolph Titramax 1000 rotary shaker (Heidolph, Schwalbach, Germany) with 1100 rpm at 42 °C. The OD_600_ was determined using the microtiter plate photometer Spectramax 340 (Molecular Devices, Ismaning, Germany) at the time points indicated in the respective Figures. Three biological replicates were performed, and average values and standard deviations were calculated.

### 2.4. Analysis of the Sensitivity to Bile Acids

Haloarchaea are very sensitive to bile acids [34,35,36]. A mixture of the sodium salts of 50% cholic acid and 50% deoxycholic acid (Honeywell Fluka; No. 48305) was used to test the sensitivity of *H. volcanii* to bile acids and to optimize the concentration. The wild-type strain H26 was grown in the presence of a various concentration of bile acids in the synthetic medium with casamino acids as carbon and energy source. Concentrations of 0.030 mg/mL and 0.035 mg/mL were chosen for the analysis, because the former still allowed the growth of the wild-type, while the latter completely inhibited growth. The cultures were grown in microtiter plates as described above, except that the precultures did not contain bile acids and thus did not have exactly the same conditions as the test cultures.

### 2.5. Swarm Plate Assays

The swarming assays were performed in six well plates (Sarstedt, Nümbrecht, Germany). Each well was filled with 5 mL synthetic medium with glucose as carbon and energy source and a reduced agar concentration of 0.3% (*w*/*v*) one day prior to their usage. Cultures were grown in glucose medium to the mid-exponential growth phase. The OD_600_ was determined, aliquots were pelleted by centrifugation, and the cell pellets were suspended in basal salts (medium without carbon source) to yield an OD_600_ of 20.2 µL of cell suspension, which was injected deep into the semi-liquid medium, to ensure a reduced oxygen concentration for motility of *H. volcanii*, as the cells do not swarm at the surface. The plates were incubated at 42 °C in a Styrofoam box together with a glass of water to inhibit drying. Every day the plates were analyzed, pictures were taken, and the swarming diameter was determined. Three biological replicates were performed, and averages and standard deviations were calculated. The results were normalized to the wild-type H26.

### 2.6. Analysis of Biofilm Formation

For the biofilm assay, cultures were grown in synthetic medium with glucose to the mid-exponential growth phase. The OD_600_ was measured, cells were pelleted by centrifugation and resuspended in fresh medium to an OD_600_ of 0.5. For biofilm formation, 96 well flat base microtiter plates were used (Sarstedt, Nümbrecht, Germany). The biofilm assay consists of several steps, i.e., formation of a biofilm, removal of planktonic cells, fixation and staining of adherent cells, and destaining and photometric quantification of the supernatant. The assay has been performed as described previously by Legerme et al. [37], with a few modifications. To this end, 150 µL of cell suspensions were pipetted in each well, and the plates were incubated without shaking at 42 °C for 24 h or 48 h. After that, the supernatant was removed, and 200 µL of fixing solution (2% (*w*/*v*) acetic acid) was given into each well, and the plate was incubated for 10 min at room temperature. The supernatant was removed, and the plate was dried for 10 min at 37 °C. After that, 200 µL staining solution (0.1% (*w*/*v*) crystal violet) was given into each well, and it was incubated for 10 min at room temperature. The supernatant was removed, and the wells were washed three times with 200 µL distilled water, respectively. After that, 200 µL of destaining solution (10% (*v*/*v*) acetic acid, 30% (*v*/*v*) methanol) was given into each well, and the plate was incubated for 10 min at room temperature. The supernatant was transferred into a new microtiter plate, and the OD_600_ was recorded with a microtiter plate photometer (Spectramax 340, Molecular Devices, San Jose, CA, USA). Three biological replicates with six technical replicates each were performed, and average values and standard deviations were calculated. A negative control (medium without cells) was included in the assay, and its value (about 0.05) was subtracted from the values of all tested strains.

### 2.7. Databases and Bioinformatics Analyses

Bioinformatic analyses of the *H. volcanii* genome were performed at the website Halolex [38]. The Halolex database is freely available, but currently usage is restricted to registered users. To request access, send a mail to halolex@rzg.mpg.de. The Integrated Genome Browser [39] was used to visualize the genome annotation, as well as the results of the dRNA-Seq study [40] and a recent RNA-Seq study [41].

## 3. Results

### 3.1. Selection of Genes and Generation of In Frame Deletion Mutants

The annotated proteins of *H. volcanii* were retrieved from the Halolex genome database and sorted according to their predicted size. Table 1 summarizes the numbers of small proteins that are predicted to be present in the size classes from 40 aa to 100 aa, all of which are missed in standard genome annotations.

Only 80 proteins are extremely small with a length of up to 50 aa, however, the number of proteins with a size of up to 100 aa is much higher and reaches 575 proteins. This accounts for about 13% of all annotated proteins of *H. volcanii*, and thus small proteins represent a considerable fraction of the *H. volcanii* proteome.

Similar to most if not all other prokaryotic and eukaryotic species, small proteins are understudied in *H. volcanii*. Only three out of eighty proteins of up to 50 aa, and only 72 of 575 proteins of up to 100 aa have an annotated biological function.

A subgroup of the small proteins contains two C(P)XCG motifs and is thus comprised of putative zinc finger proteins. The fraction is higher in very small proteins of up to 50 aa (21%) as in all small proteins of up to 100 aa (12%). While 69 C(P)XCG zinc finger proteins have a length of up to 100 aa, only 16 are longer than 100 aa. Only four are longer than 150 aa, and the largest has a length of 172 aa. Therefore, all 85 C(P)XCG zinc finger proteins of *H. volcanii* are considerably smaller than the average protein size of about 300 aa. None of the C(P)XCG proteins has a predicted annotated function, and thus these putative single domain zinc finger proteins are of specific novelty. Therefore, we aimed to reveal whether or not C(P)XCG µ-proteins have important or essential roles for *H. volcanii*, and to elucidate their participation at specific biological functions.

43 of the C(P)XCG µ-proteins have a length of up to 70 aa, the maximal length that can be studied in the framework of the German Priority Program 2002 “Small proteins in prokaryotes: An unexplored words” (http://www.spp2002.uni-kiel.de). Therefore, the genes for this study had to be selected from this set of 43 proteins. The experimental design was comprised of the generation of in frame deletion mutants and of the phenotypic comparison of wild-type and mutants under various conditions. Sixteen genes were selected for this study based on the following criteria: (1) The identity of the neighboring genes, with an emphasis on genes with known functions; (2) the expression level under optimal conditions, derived from the number of reads determined in a recent dRNA-Seq study [40]; (3) the cysteine content (with an emphasis on proteins that do not contain cysteines outside of the two motifs); and (4) the isoelectric point (with the aim to include proteins with acidic, as well as basic pIs). Supplementary Table S2 gives an overview of the characteristics of the 16 selected proteins.

A few years ago we have adapted many steps of the workflow for deletion mutant generation to the application of microtiter plates [29], e.g., PCRs, fusion PCRs, cloning. This parallel approach facilitates and accelerates the construction of a double-digit number of in frame deletion mutants, without the need of any robotics, and it is, therefore, well suited for a small group in a University setting. The oligonucleotides used to construct the 16 deletion plasmids are summarized in Supplementary Table S1. The sequences of the plasmids were verified by sequencing, and the plasmids were used to transform *H. volcanii* strain H26 [26]. The deletion mutants were selected using the so called Pop-In-Pop-Out method [29,42]. *H. volcanii* is polyploid [31], therefore, great care has to be taken to ensure that all genome copies contain the deletion. The homozygocities of the deletion mutants were verified using PCR, as well as Southern blot analyses. Supplementary Table S2 summarizes the genomic coordinates of the 16 deleted regions. All 16 homozygous deletion mutants could readily be generated, showing that none of the 16 proteins is essential for *H. volcanii.* All 16 mutants had the same colony size and colony form as the wild-type. In addition, all colonies developed the same red color, showing that all mutants were proficient in carotenoid biosynthesis. The subsequent phenotypic analyses aimed at testing many different biological functions and pathways.

### 3.2. Growth Analyses in Media with Different Carbon and Phosphate Sources

**Figure 1:**
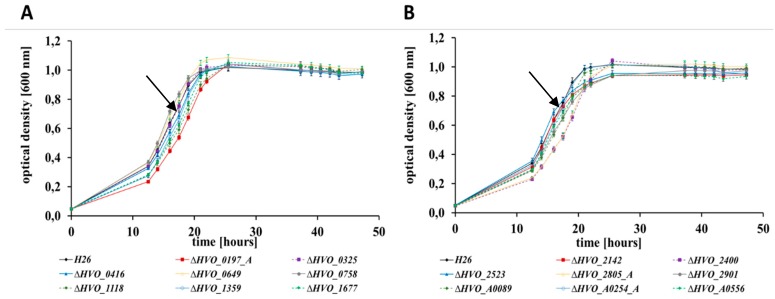
Growth curves of the parent strain *H26* (black) and 16 deletion mutants (in color) grown in a complex medium. (**A**) Parent strain H26 (black) and the deletion mutants ∆*0197_A* (red), ∆*0325* (purple), ∆*0416* (dark blue), ∆*0649* (yellow), ∆*0758* (grey), ∆*1118* (dark green), ∆*1359* (light blue), ∆*1677* (light green). (**B**) Parent strain H26 (black) and the deletion mutants ∆*2142* (red), ∆*2400* (purple), ∆*2523* (dark blue), ∆*2805_A* (yellow), ∆*2901* (grey), ∆*A0089* (dark green), ∆*A0254_A* (light blue), ∆*A0556* (light green). The wild-type is indicated by black arrows.

**Figure 2:**
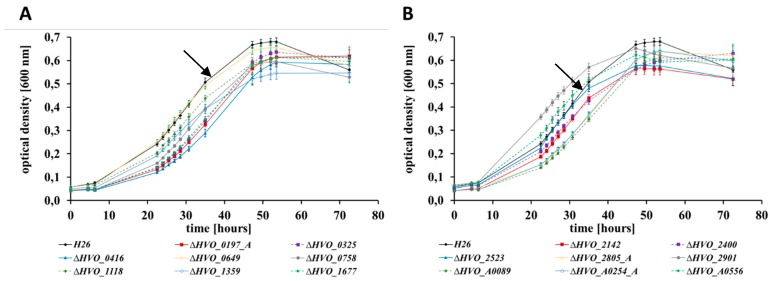
Growth curves of the parent strain *H26* (black) and 16 deletion mutants (in color) grown in synthetic medium with glucose as carbon source. (**A**) Parent strain H26 (black) and deletion mutants ∆*0197_A* (red), ∆*0325* (purple), ∆*0416* (dark blue), ∆*0649* (yellow), ∆*0758* (grey), ∆*1118* (dark green), ∆*1359* (light blue), ∆*1677* (light green). (**B**) Parent strain H26 and deletion mutants ∆*2142* (red), ∆*2400* (purple), ∆*2523* (dark blue), ∆*2805_A* (yellow), ∆*2901* (grey), ∆*A0089* (dark green), ∆*A0254_A* (light blue), ∆*A0556* (light green). The wild-type is indicated by black arrows.

**Figure 3:**
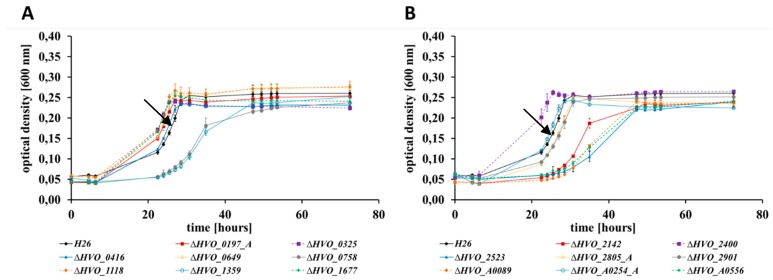
Growth curves of the parent strain *H26* (black) and 16 deletion mutants (in color) grown in synthetic medium with glycerol as carbon source. (**A**) Parent strain H26 (black) and deletion mutants ∆*0197_A* (red), ∆*0325* (purple), ∆*0416* (dark blue), ∆*0649* (yellow), ∆*0758* (grey), ∆*1118* (dark green), ∆*1359* (light blue), ∆*1677* (light green). (**B**) Parent strain H26 (black) and deletion mutants ∆*2142* (red), ∆*2400* (purple), ∆*2523* (dark blue), ∆*2805_A* (yellow), ∆*2901* (grey), ∆*A0089* (dark green), ∆*A0254_A* (light blue), ∆*A0556* (light green). The wild-type is indicated by black arrows.

**Figure 4:**
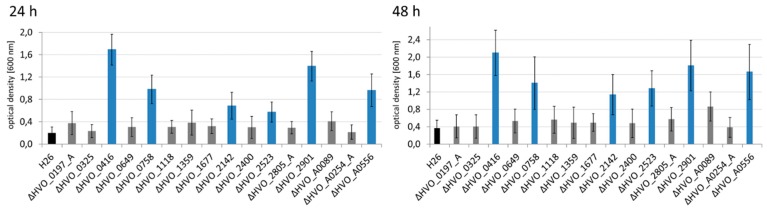
Comparison of the biofilm formation of the parent strain H26 (black) and its 16 deletion mutants after 24 h and 48 h incubation. The mutants with a gain of function phenotypes are shown in blue. Average values of three biological replicates and their standard deviations are shown.

At first, the wild-type and the 16 in frame deletion mutants were grown in a complex medium at 42 °C with good aeration, and growth curves were recorded (Figure 1). The growth of all 17 strains was nearly identical, indicating that the 16 CPXCP µ-proteins have no important roles under optimal conditions. Growth of the wild-type and the 16 mutants were also monitored in synthetic medium with casamino acids as carbon source. This condition did not require any amino acid biosynthesis, but catabolism of amino acids and anabolism of sugars. Again, none of the mutants exhibited a growth defect (Supplementary Figure S1). Next, growth in synthetic medium with glucose as the sole carbon and energy source was tested (Figure 2). This condition required the catabolism of glucose and the biosynthesis of all 20 amino acids. In this medium, very slight differences between the mutants and the wild-type could be observed. One mutant grew slightly better than the wild-type (dHVO_2901), two mutants grew indistinguishable from the wild-type (dHVO_A0556), and the remaining 13 mutants had very minor growth defects. The next tested carbon and energy source was glycerol (Figure 3). Glycerol is a preferred carbon source for *H. volcanii* and in fact represses glucose metabolism [43]. Unexpectedly, there was a difference between glycerol and the other carbon sources, and six of the mutants exhibited a severe growth defect (Figure 4). Again, one deletion mutant grew better than the wild-type (dHVO_2400).

*H. volcanii* can use external, environmental genomic DNA as a phosphate source [33]. Therefore, it was tested whether this ability was affected by any of the deletion mutants, using a synthetic medium with genomic DNA as phosphate source and casamino acids as carbon and energy source (Supplementary Figure S2). It turned out that all 16 deletion mutants grew indistinguishable from the wild-type. Taken together, severe growth defects were observed in only one of five different media tested, i.e., in synthetic medium with glycerol as carbon and energy source.

### 3.3. Sensitivities to Bile Acids

*Haloferax* and several additional genera of haloarchaea are very sensitive to bile acids, and even moderate concentrations result in cell lysis [34,35,36]. It can be expected that the degree of sensitivity is correlated with the lipid composition of the membrane, and, thus, it might well be an indicator of lipid metabolism. Therefore, it was tested whether the degree of sensitivity of wild-type and deletion mutants was identical, or whether differences exist. At first, various concentrations were used in casamino acids medium to characterize the sensitivity of the wild-type. It was verified that *H. volcanii* is very sensitive to bile acids, and, it was also found, that very small concentration differences resulted in considerable growth differences. Concentrations of 0.030 mg/mL and 0.035 mg/mL were chosen for further experiments, because the wild-type grew rather well at the former concentration, while it was totally inhibited by the latter concentration. The growth curves of the wild-type and all deletion mutants at both concentrations are shown in Supplementary Figures S3 and S4. The variances between the biological replicates were extremely large, in contrast to all other growth analyses. The reason is most probably that already small differences in evaporation result in concentration changes that affect growth. Therefore, the results have to be treated with care, and only a qualitative analysis seems possible. However, despite the high variances, consistent differences between the wild-type and several mutants were observed at consecutive time points. Four mutants were severely inhibited by 0.030 mg/mL, in contrast to the wild-type and the other mutants (Supplementary Figure S3A, highlighted with a red bar). In contrast, four other mutants showed considerable growth in the presence of 0.035 mg/mL, in contrast to all other strains (Supplementary Figure S4B, highlighted by a red bar). Taken together, the analysis of bile acids sensitivity demonstrated large differences between wild-type and eight deletion mutants. To our knowledge, this is the first time that this assay was used for phenotypic mutant analysis in haloarchaea.

### 3.4. The Different Life Styles of H. volcanii: Swarming and Biofilm Formation

*H. volcanii*, like many other haloarchaeal species, can form biofilms [44]. It can be expected that many different gene products are essential for biofilm formation. In fact, more than 10 adhesion mutants were identified using a transposon mutagenesis screen [37]. The principle of the biofilm assay is to incubate a culture to allow the formation of a biofilm, remove planktonic cells, and fix and stain adherent cells [37]. The amount of dye bound to a biofilm correlates with its size, and thus, after destaining, photometric analysis of the dye yields a quantitative value for biofilm formation. The quantification of biofilm formation after 24 h and after 48 h is shown in Figure 4. None of the mutants had a defect in biofilm formation. In stark contrast, six of the mutants exhibited a several fold increase in biofilm formation, from threefold to ninefold (blue columns in Figure 4). Biofilm formation of the remaining ten mutants was very similar to that of the wild-type.

**Figure 5:**
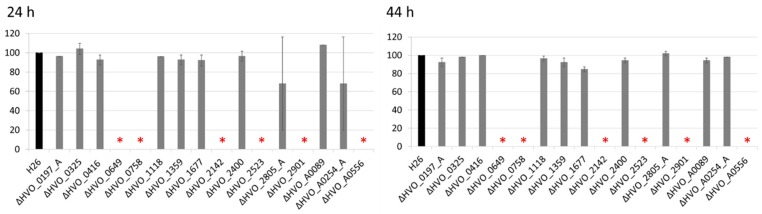
Comparison of the swarming velocity of the parent strain H26 (black) and its 16 deletion mutants after 24 h and 44 h incubation. Average values of three biological replicates and their standard deviations are shown. At both time points, the swarming diameters of the mutants were normalized to that of the parent strain H26 (= 100%). The wild-type had a swarming diameter of 5 mm after 24 h and 23 mm after 48 h. The cultures were injected into the semi-solid agar using a tip with 1 mm diameter. Colonies with no observable swarming with a colony diameter of less than 2 mm after 44 h were regarded to have a null phenotype. The mutants with a null phenotype are indicated by red asterisks.

Since its discovery in 1975 *H. volcanii* was regarded to be non-motile [45]. However, a few years ago a swarm plate assay was developed, which revealed that *H. volcanii* can swim and shows chemotaxis under conditions of reduced oxygen concentrations [46]. The assay was applied to quantify swarming velocity of wild-type and deletion mutants after 24 h and 44 h (Figure 5). Six mutants exhibited a null phenotype and had completely lost the ability to swarm (red asterisks in Figure 5. Remarkably, five of the six mutants with a swarm defect concomitantly showed increased biofilm formation (compare Figure 4; Figure 5). The two phenotypes were uncoupled in only two mutants, i.e., mutant dHVO_0416 showed only increased biofilm formation, and mutant dHVO_0649 had solely a swarm defect. Together, the results show a high anti-correlation between a motile and a sessile lifestyle of *H. volcanii*, but in addition, that both life styles can also be affected individually.

## 4. Discussion

Until now, only very few archaeal µ-proteins of less than 70 aa have an annotated function and have been experimentally characterized. Many of these are small subunits of large complexes, e.g., eight subunits of the ribosome belong to this group (Rps14/17e and Rpl20e/24e/29/37e/39e/40e), as well as the subunits RpoK and RpoP of RNA polymerase. Very early, a small DNA binding protein of 64 aa was isolated from cell extracts of *Sulfolobus solfataricus* using classical biochemical approaches [47]. Recently, five µ-proteins from *Methanosarcina mazei* Gö1 were identified by LC-MS/MS, which had sizes from 23 aa to 61 aa [48]. The transcript levels of two of the respective genes were severely decreased in stationary phase, while one was increased 2.5-fold in response to nitrogen limitation. Overproduction of the three proteins, respectively, resulted in changes in transcript levels of 10–20 genes, but, unfortunately, did not result in any phenotypic changes, which could reveal important functions [48]. In *H. volcanii* three “small archaeal modifier proteins (SAMPs)” have been identified, which were first described to modify proteins analogous to the eukaryotic ubiquitin and are thus important for protein degradation in the proteasome [49]. All three are small proteins with less than 100 aa. However, only SAMP2 with 66 aa fulfills the definition of a µ-protein, while SAMP1 has 87 aa and SAMP3 has 92 aa. Later, it was found that the SAMPs are not only involved in protein degradation, but also in sulfur metabolism and oxidative stress response [50,51].

The biological functions of a few additional *H. volcanii* µ-proteins have been studied, however, only 24 of the 282 annotated µ-protein genes have a known function (Table 1). Much more dramatically, none of the 43 putative zinc finger µ-proteins has a known function. Therefore, this group was chosen for this study, with the aim to reveal whether or not they have important biological roles. To our knowledge, prior to our study only one single putative zinc finger µ-protein gene has been studied in any prokaryotic or eukaryotic species, i.e., the *brz* gene of *H. salinarum* [22]. Brz contains a C3H zinc finger with one C(P)XCG motif and one CXXXH motif. Replacement of the second cysteine in the first motif and the histidine in the second motif resulted in the same drastic decrease of the *bop* mRNA level as the deletion of the whole gene, showing that both motifs are indispensable for function.

We chose 16 of the 43 putative zinc finger µ-proteins of *H. volcanii* for analysis, based on the identity of adjacent genes, transcript levels under optimal conditions, pI values, and cysteine content. In frame deletion mutants of all 16 genes could readily be generated, showing that none of the genes is essential. Furthermore, colony size, colony form, and colony color were identical for all mutants and the wild-type, which already indicated that the mutants did not have a severe growth defect in complex medium. The mutants were compared to the wild-type under eight different conditions, which should involve various metabolic pathways and the genes serving them. The results are summarized in Table 2. Notably, none of the mutants exhibited a growth phenotype under optimal or near-optimal conditions. In contrast, growth of eight of the 16 mutants differed from the wild-type in the presence of bile acids, which represent a strong stressor for *H. volcanii*. In total, 12 of the 16 deletion mutants showed a changed phenotype under at least one conditions, underscoring the high importance of the zinc finger µ-proteins. Seven mutants had a phenotype under two or more conditions, and they were thus pleiotropic. About the same number were gain of function and loss of function mutants, indicating that zinc finger µ-proteins are members of regulatory networks that take many environmental conditions into account. The diversity of phenotypes indicates that the functions of the proteins are not uniform or similar. Notably, three deletion mutants had a pleiotropic phenotype under four rather different conditions, which might indicate that the respective zinc finger µ-proteins have a high level in regulatory hierarchies and influence other regulators.

Remarkably, the deletion of each of the disclosed seven genes had an influence on the life styles “biofilm formation” and “swarming” of *H. volcanii*. In five cases the deletions led to an increase in biofilm formation and a concomitant decrease in swarming. Therefore, it seems that these zinc finger µ-proteins promote the motile lifestyle and counteract a sessile lifestyle.

Decision making regulatory circuits between a motile and a sessile lifestyle have also been reported for several bacteria, e.g., *Bacillus subtilis, Escherichia coli*, *Pseudomonas aeruginosa*, and *Vibrio cholera* [52]. In bacteria, the signaling molecule c-di-GMP is involved, i.e., high concentrations of c-di-GMP correlate with a high probability of a sessile lifestyle and a low probability of a motile lifestyle [53]. However, archaea do not use c-di-GMP, and thus the molecular mechanism of sessile versus motile decisions must be different in *H. volcanii*.

It seems that also small non-coding RNAs (sRNAs) are involved, because six sRNA gene deletion mutants of *H. volcanii* had a swarming phenotype [29]. Biofilm formation was not addressed in this previous study [29], therefore, it is not known whether the opposite regulation described above for five zinc finger µ-proteins holds true for sRNAs in *H. volcanii*.

The influence of sRNAs on biofilm formation and swarming has also been analyzed in *E. coli* [54]. Ninty-nine sRNAs were overexpressed, and the effects on several traits were quantified. While the overexpression of many sRNAs influenced biofilm formation, swarming, or both, no consistent regulatory pattern could be observed. For example, six sRNAs had an opposing effect on the sessile and motile lifestyle, while, in contrast, eight effected the life styles in the same direction. Biofilm formation and swarming are very complex traits, therefore, complex patterns can be expected, which involve regulators at different levels, as well as structural proteins.

For *H. volcanii*, important components in addition to µ-proteins and sRNAs have been revealed: A transposon mutagenesis screening led to the identification of 17 motility mutants and 11 adhesion mutants, without any overlap between the two groups [37]. Most of the inactivated genes did not have an obvious connection to the motility of biofilm formation, underscoring the complexity of these two traits. Swarming and biofilm formation has already been studied to some extent in *H. volcanii*. For example, it has been shown that flagella are required for swarming, but not for surface adhesion [46]. On the other hand, it was revealed that pili are essential for surface adhesion [55,56]. Confocal scanning microscopy was used to demonstrate the complex morphological development of biofilms over two days [57]. However, much has to be learned about the involved regulatory networks and structural proteins mediating morphological changes. The seven zinc finger µ-proteins described above are unexpected new players in the game.

The phenotypic differences between 12 deletion mutants and the wild-type (Table 2) do not yet yield insight into the regulatory networks and molecular mechanisms of haloarchaeal zinc finger µ-proteins. However, besides the single example of *brz* of *H. salinarum* [22], they represent the first experimental proof in any prokaryotic or eukaryotic species for the high biological importance of zinc finger µ-proteins. The clear phenotypes will pave the way for future in depth characterizations. The next step will be the attempt to complement the mutants with tagged versions of the respective native proteins, which would allow co-affinity purification strategies to reveal interaction networks. This will probably not be easy, because the addition of tags often perturbs the functions of µ-proteins [1], and the number of tags that are compatible with the high salt cytoplasm of haloarchaea is small. In any case, also complementation with the untagged native versions will be informative, because it will allow to test point mutated versions and address the importance of single amino acids for protein function. In conclusion, this study demonstrated that various members of the family of zinc finger µ-proteins, which with one exception has not been studied until now, have important roles in stress adaptation (at least membrane stress) and life style decisions in *H. volcanii*.

## Figures and Tables

**Table 1:** Length distribution of small proteins encoded in the genome of *H. volcanii*.

Length up to aa	All Proteins			C(P)XCG Motif Proteins		
	No.	Annotated Function	(%)	No.	(%)	Annotated Function
40	27	0	0	3	11	0
50	80	3	4	17	21	0
60	167	10	6	36	22	0
70	282	24	9	43	15	0
80	373	30	8	56	15	0
90	468	49	10	61	13	0
100	575	72	13	69	12	0

**Table 2:** Summary of phenotypic analysis of 16 zinc finger gene deletion mutants. “-” indicates that wild-type and deletion mutant are indistinguishable, “loss” indicates a loss of function phenotype of the mutant, “gain” a gain of function phenotype. Three mutants with the same pleiotrophic phenotype are shown in red.

Deletion Mutant	Growth in Four Media ^*1^	Growth on Glycerol	Bile Acid ^*2^	Bio-Film	Swarming	Pattern	No. of Phenotypes
HVO_0197_A	-	-	(loss)	-	-		1
HVO_0325	-	-	-	-	-		0
HVO_0416	-	-	-	gain	-		1
HVO_0649	-	-	(loss)	-	loss		2
HVO_0758	-	loss	-	gain	loss	(Yes)	3
HVO_1118	-	-	(loss)	-	-		1
HVO_1359	-	loss	(loss)	-	-		2
HVO_1677	-	-	-	-	-		0
HVO_2142	-	loss	(gain)	gain	loss	Yes	4
HVO_2400	-	gain	-	-	-		1
HVO_2523	-	loss	(gain)	gain	loss	Yes	4
HVO_2805_A	-	-	-	-	-		0
HVO_2901	-	-	(gain)	gain	loss	(Yes)	3
HVO_A0089	-	loss	-	-	-		1
HVO_A0254_A	-	-	-	-	-		0
HVO_A0556	-	loss	(gain)	gain	loss	Yes	4
sum	0	7	8	6	6		

^*1^ growth in complex medium, synthetic medium with casamino acids and with glucose as carbon source, synthetic medium with genomic DNA as phosphate source ^*2^ the growth in the presence of bile acids had a very high variance. Therefore, the results should be handled with care and they are shown in brackets.

## Supplementary Materials

The following are available online at https://www.mdpi.com/2073-4425/10/5/361/s1, Table S1: List of primers used for the generation of deletion mutants. Table S2: Overview of characteristics of the analyzed 16 zinc finger proteins. Figures S1–S4: Growth in synthetic medium with casamino acids, growth with genomic DNA as phosphate source, growth in the presence of 0.030 mg/mL and 0.035 mg/mL bile acids.

## References

[1] Duval M., Cossart P. (2017). Small bacterial and phagic proteins: An updated view on a rapidly moving field. Curr. Opin. Microbiol..

[2] Baumgartner D., Kopf M., Klähn S., Steglich C., Hess W.R. (2016). Small proteins in cyanobacteria provide a paradigm for the functional analysis of the bacterial micro-proteome. BMC Microbiol..

[3] Storz G., Wolf Y.I., Ramamurthi K.S. (2014). Small proteins can no longer be ignored. Annu. Rev. Biochem..

[4] Delcourt V., Staskevicius A., Salzet M., Fournier I., Roucou X. (2018). Small proteins encoded by unannotated ORFs are rising stars of the proteome, confirming shortcomings in genome annotations and current vision of an mRNA. Proteomics.

[5] Plaza S., Menschaert G., Payre F. (2017). In Search of lost small peptides. Annu. Rev. Cell Dev. Biol..

[6] Cabrera-Quio L.E., Herberg S., Pauli A. (2016). Decoding sORF translation—From small proteins to gene regulation. RNA Biol..

[7] Hao Y., Zhang L., Niu Y., Cai T., Luo J., He S., Zhang B., Zhang D., Qin Y., Yang F. (2018). SmProt: A database of small proteins encoded by annotated coding and non-coding RNA loci. Brief. Bioinform..

[8] Olexiouk V., van Criekinge W., Menschaert G. (2018). An update on sORFs.org: A repository of small ORFs identified by ribosome profiling. Nucleic Acids Res..

[9] Mumtaz M.A.S., Couso J.P. (2015). Ribosomal profiling adds new coding sequences to the proteome. Biochem. Soc. Trans..

[10] Schrader M. (2018). Origins, technological development, and applications of peptidomics. Methods Mol. Biol..

[11] Klein C., Aivaliotis M., Olsen J.V., Falb M., Besir H., Scheffer B., Bisle B., Tebbe A., Konstantinidis K., Siedler F. (2007). The low molecular weight proteome of *Halobacterium salinarum*. J. Proteome Res..

[12] Maret W. (2013). Zinc biochemistry: From a single zinc enzyme to a key element of life. Adv. Nutr..

[13] Cassandri M., Smirnov A., Novelli F., Pitolli C., Agostini M., Malewicz M., Melino G., Raschellà G. (2017). Zinc-finger proteins in health and disease. Cell Death Discov..

[14] Eom K.S., Cheong J.S., Lee S.J. (2016). Structural analyses of zinc finger domains for specific interactions with DNA. J. Microbiol. Biotechnol..

[15] Matthews J.M., Sunde M. (2002). Zinc fingers—Folds for many occasions. IUBMB Life.

[16] Krishna S.S., Majumdar I., Grishin N.V. (2003). Structural classification of zinc fingers: Survey and summary. Nucleic Acids Res..

[17] Chou A.Y., Archdeacon J., Kado C.I. (1998). *Agrobacterium* transcriptional regulator Ros is a prokaryotic zinc finger protein that regulates the plant oncogene IPT. Proc. Natl. Acad. Sci. USA.

[18] Bouhouche N., Syvanen M., Kado C.I. (2000). The origin of prokaryotic C2H2 zinc finger regulators. Trends Microbiol..

[19] Malgieri G., Palmieri M., Russo L., Fattorusso R., Pedone P.V., Isernia C. (2015). The prokaryotic zinc-finger: Structure, function and comparison with the eukaryotic counterpart. FEBS J..

[20] Pereira L.E., Tsang J., Mrázek J., Hoover T.R. (2011). The zinc-ribbon domain of *Helicobacter pylori* HP0958: Requirement for RpoN accumulation and possible roles of homologs in other bacteria. Microb. Inform. Exp..

[21] Weidenbach K., Ehlers C., Schmitz R.A. (2014). The transcriptional activator NrpA is crucial for inducing nitrogen fixation in *Methanosarcina mazei* Gö1 under nitrogen-limited conditions. FEBS J..

[22] Tarasov V.Y., Besir H., Schwaiger R., Klee K., Furtwängler K., Pfeiffer F., Oesterhelt D. (2008). A small protein from the *bop-brp* intergenic region of *Halobacterium salinarum* contains a zinc finger motif and regulates *bop* and *crtB1* transcription. Mol. Microbiol..

[23] Soppa J. (2006). From genomes to function: Haloarchaea as model organisms. Microbiology.

[24] Pohlschroder M., Schulze S. (2019). *Haloferax* *volcanii*. Trends Microbiol..

[25] Pfeiffer F., Oesterhelt D. (2015). A manual curation strategy to improve genome annotation: Application to a set of haloarchael genomes. Life.

[26] Allers T., Ngo H.-P., Mevarech M., Lloyd R.G. (2004). Development of additional selectable markers for the halophilic archaeon *Haloferax volcanii* based on the *leuB* and *trpA* genes. Appl. Environ. Microbiol..

[27] Dambeck M., Soppa J. (2008). Characterization of a *Haloferax volcanii* member of the enolase superfamily: Deletion mutant construction, expression analysis, and transcriptome comparison. Arch. Microbiol..

[28] Green M.R., Sambrook K. (2012). Molecular Cloning: A Laboratory Manual.

[29] Jaschinski K., Babski J., Lehr M., Burmester A., Benz J., Heyer R., Dörr M., Marchfelder A., Soppa J. (2014). Generation and phenotyping of a collection of sRNA gene deletion mutants of the haloarchaeon *Haloferax volcanii*. PLoS ONE.

[30] Hammelmann M., Soppa J. (2008). Optimized generation of vectors for the construction of *Haloferax volcanii* deletion mutants. J. Microbiol. Methods.

[31] Breuert S., Allers T., Spohn G., Soppa J. (2006). Regulated polyploidy in halophilic archaea. PLoS ONE.

[32] Jantzer K., Zerulla K., Soppa J. (2011). Phenotyping in the archaea: Optimization of growth parameters and analysis of mutants of *Haloferax volcanii*. FEMS Microbiol. Lett..

[33] Zerulla K., Chimileski S., Näther D., Gophna U., Papke R.T., Soppa J. (2014). DNA as a phosphate storage polymer and the alternative advantages of polyploidy for growth or survival. PLoS ONE.

[34] Kamekura M., Oesterhelt D., Wallace R., Anderson P., Kushner D.J. (1988). Lysis of halobacteria in bacto-peptone by bile acids. Appl. Environ. Microbiol..

[35] Elevi Bardavid R., Oren A. (2008). Sensitivity of *Haloquadratum* and *Salinibacter* to antibiotics and other inhibitors: Implications for the assessment of the contribution of Archaea and Bacteria to heterotrophic activities in hypersaline environments. FEMS Microbiol. Ecol..

[36] Kumar V., Saxena J., Tiwari S.K. (2016). Description of a halocin-producing *Haloferax larsenii* HA1 isolated from Pachpadra salt lake in Rajasthan. Arch. Microbiol..

[37] Legerme G., Yang E., Esquivel R.N., Kiljunen S., Savilahti H., Pohlschroder M. (2016). Screening of a *Haloferax volcanii* transposon library reveals novel motility and adhesion mutants. Life.

[38] Pfeiffer F., Broicher A., Gillich T., Klee K., Mejía J., Rampp M., Oesterhelt D. (2008). Genome information management and integrated data analysis with HaloLex. Arch. Microbiol..

[39] Freese N.H., Norris D.C., Loraine A.E. (2016). Integrated genome browser: Visual analytics platform for genomics. Bioinformatics.

[40] Babski J., Haas K.A., Näther-Schindler D., Pfeiffer F., Förstner K.U., Hammelmann M., Hilker R., Becker A., Sharma C.M., Marchfelder A. (2016). Genome-wide identification of transcriptional start sites in the haloarchaeon *Haloferax volcanii* based on differential RNA-Seq (dRNA-Seq). BMC Genom..

[41] Laass S., Monzon V.A., Kliemt J., Hammelmann M., Pfeiffer F., Förstner K.U., Soppa J. (2019). Characterization of the transcriptome of *Haloferax volcanii*, grown under four different conditions, with mixed RNA-Seq. PLoS ONE.

[42] Bitan-Banin G., Ortenberg R., Mevarech M. (2003). Development of a gene knockout system for the halophilic archaeon *Haloferax volcanii* by use of the *pyrE* gene. J. Bacteriol..

[43] Sherwood K.E., Cano D.J., Maupin-Furlow J.A. (2009). Glycerol-mediated repression of glucose metabolism and glycerol kinase as the sole route of glycerol catabolism in the haloarchaeon *Haloferax volcanii*. J. Bacteriol..

[44] Fröls S., Dyall-Smith M., Pfeifer F. (2012). Biofilm formation by haloarchaea. Environ. Microbiol..

[45] Mullakhanbhai M.F., Larsen H. (1975). *Halobacterium volcanii* spec. nov., a Dead Sea halobacterium with a moderate salt requirement. Arch. Microbiol..

[46] Tripepi M., Imam S., Pohlschröder M. (2010). *Haloferax volcanii* flagella are required for motility but are not involved in PibD-dependent surface adhesion. J. Bacteriol..

[47] Kimura M., Kimura J., Davie P., Reinhardt R., Dijk J. (1984). The amino acid sequence of a small DNA binding protein from the archaebacterium *Sulfolobus solfataricus*. FEBS Lett..

[48] Prasse D., Thomsen J., de Santis R., Muntel J., Becher D., Schmitz R.A. (2015). First description of small proteins encoded by spRNAs in *Methanosarcina mazei* strain Gö1. Biochimie.

[49] Humbard M.A., Miranda H.V., Lim J.-M., Krause D.J., Pritz J.R., Zhou G., Chen S., Wells L., Maupin-Furlow J.A. (2010). Ubiquitin-like small archaeal modifier proteins (SAMPs) in *Haloferax volcanii*. Nature.

[50] Dantuluri S., Wu Y., Hepowit N.L., Chen H., Chen S., Maupin-Furlow J.A. (2016). Proteome targets of ubiquitin-like samp1ylation are associated with sulfur metabolism and oxidative stress in *Haloferax volcanii*. Proteomics.

[51] Hepowit N.L., de Vera I.M.S., Cao S., Fu X., Wu Y., Uthandi S., Chavarria N.E., Englert M., Su D., Söll D. (2016). Mechanistic insight into protein modification and sulfur mobilization activities of noncanonical E1 and associated ubiquitin-like proteins of Archaea. FEBS J..

[52] Guttenplan S.B., Kearns D.B. (2013). Regulation of flagellar motility during biofilm formation. FEMS Microbiol. Rev..

[53] Verstraeten N., Braeken K., Debkumari B., Fauvart M., Fransaer J., Vermant J., Michiels J. (2008). Living on a surface: Swarming and biofilm formation. Trends Microbiol..

[54] Bak G., Lee J., Suk S., Kim D., Young Lee J., Kim K.-S., Choi B.-S., Lee Y. (2015). Identification of novel sRNAs involved in biofilm formation, motility, and fimbriae formation in *Escherichia coli*. Sci. Rep..

[55] Esquivel R.N., Schulze S., Xu R., Hippler M., Pohlschroder M. (2016). Identification of *Haloferax volcanii* Pilin N-glycans with diverse roles in pilus biosynthesis, adhesion, and microcolony formation. J. Biol. Chem..

[56] Pohlschroder M., Esquivel R.N. (2015). Archaeal type IV pili and their involvement in biofilm formation. Front. Microbiol..

[57] Chimileski S., Franklin M.J., Papke R.T. (2014). Biofilms formed by the archaeon *Haloferax volcanii* exhibit cellular differentiation and social motility, and facilitate horizontal gene transfer. BMC Biol..

