# Supplementary material for: Several One-Domain Zinc Finger µ-Proteins of Haloferax Volcanii Are Important for Stress Adaptation, Biofilm Formation, and Swarming
Source: Genes (Basel). 2019 May 10;10(5):361. doi: 10.3390/genes10050361 (PMC6562870; doi:10.3390/genes10050361)
Supplement: Supplementary file 1 [file genes-10-00361-s001.zip › Supplementary_Figures.docx]

**Supplementary Figures S1 – S4. Growth of the wildypte H26 and the 16 single *in frame* deletion mutants under various conditions.**


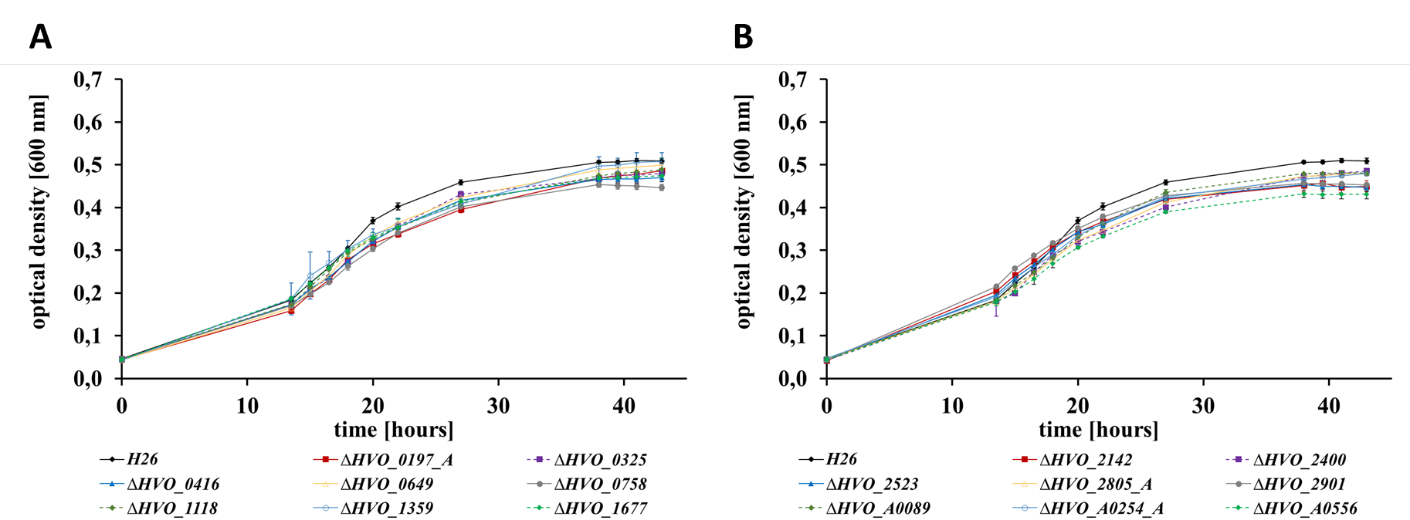


**Supplementary Figure S1.** Growth curves of the parent strain H26 (black) and 16 deletion mutants (in color) grown in synthetic medium with casamino acids as carbon source. **A.** Parent strain H26 (black) and deletion mutants *∆0197_A* (red), *∆0325* (purple), *∆0416* (dark blue), *∆0649* (yellow), *∆0758* (grey), *∆1118* (dark green), *∆1359* (light blue), *∆1677* (light green). **B.** Parent strain H26 (black) and deletion mutants *∆2142* (red), *∆2400* (purple), *∆2523* (dark blue), *∆2805_A* (yellow), *∆2901* (grey), *∆A0089* (dark green), *∆A0254_A* (light blue), *∆A0556* (light green). The wildtype is indicated by black arrows.


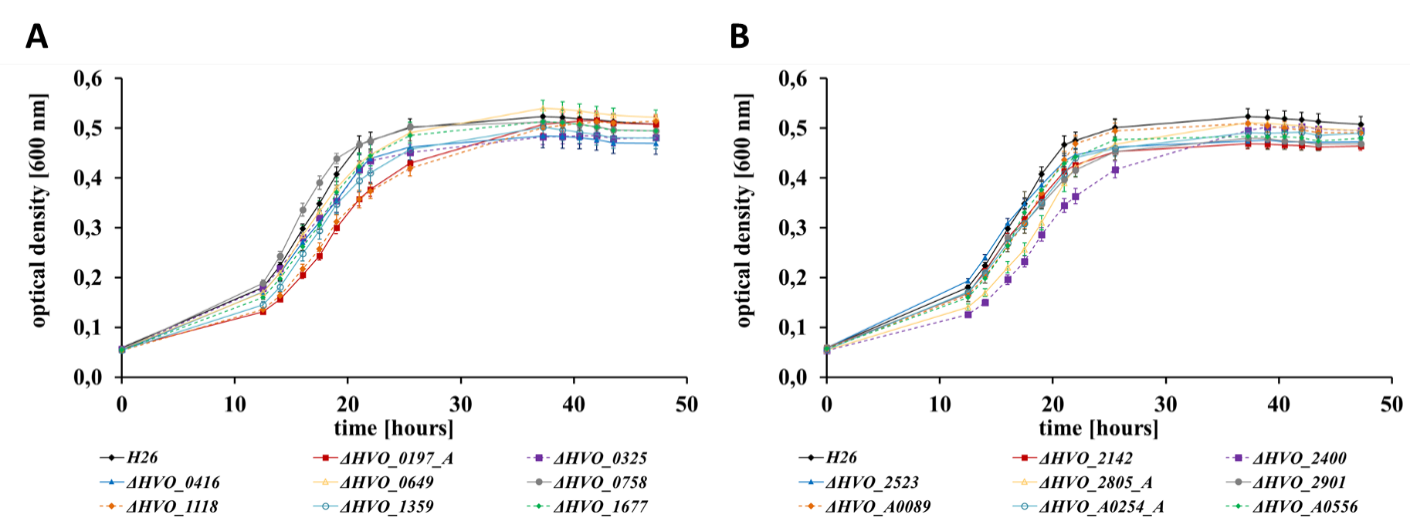


**Supplementary Figure S2**. Growth curves of the parent strain *H26* (black) and 16 deletion mutants (in color) grown in synthetic medium with *H. volcanii* genomic DNA as phosphate source. **A.** Parent strain H26 (black) and deletion mutants *∆0197_A* (red), *∆0325* (purple), *∆0416* (dark blue), *∆0649* (yellow), *∆0758* (grey), *∆1118* (dark green), *∆1359* (light blue), *∆1677* (light green). **B.** Parent strain H26 (black) and deletion mutants *∆2142* (red), *∆2400* (purple), *∆2523* (dark blue), *∆2805_A* (yellow), *∆2901* (grey), *∆A0089* (dark green), *∆A0254_A* (light blue), *∆A0556* (light green).


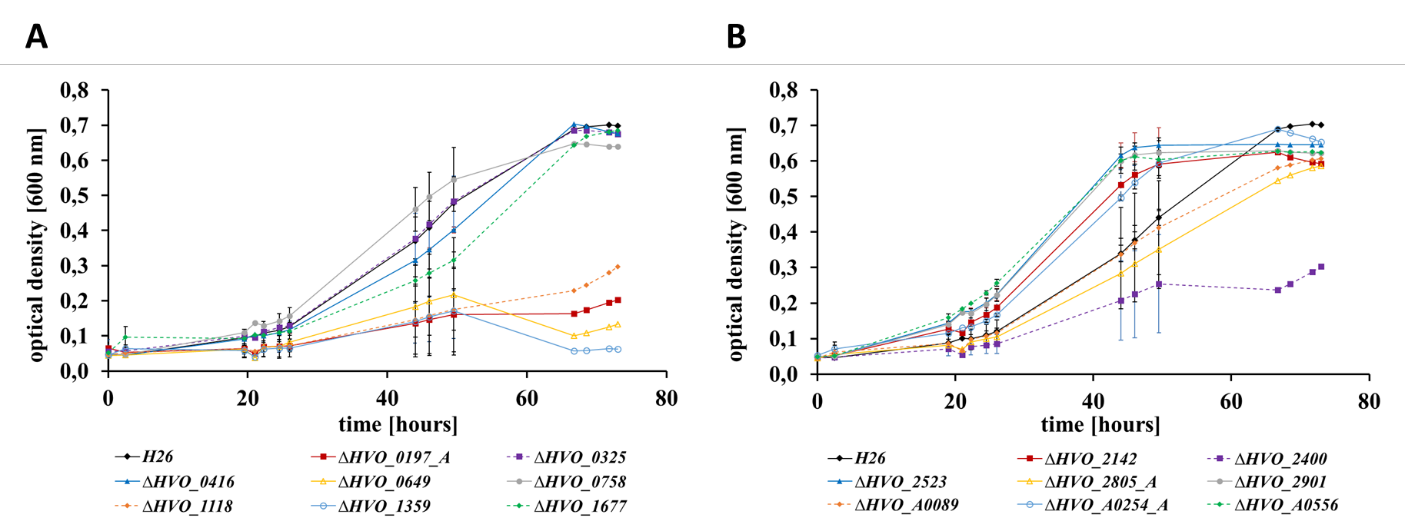


**Supplementary Figure S3**. Growth curves of the parent strain *H26* (black) and 16 deletion mutants (in color) exposed to bile acid [0.030 mg/ml]. **A.** Parent strain H26 (black) and deletion mutants *∆0197_A* (red), *∆0325* (purple), *∆0416* (dark blue), *∆0649* (yellow), *∆0758* (grey), *∆1118* (dark green), *∆1359* (light blue), *∆1677* (light green). **B.** Parent strain H26 and deletion mutants *∆2142* (red), *∆2400* (purple), *∆2523* (dark blue), *∆2805_A* (yellow), *∆2901* (grey), *∆A0089* (dark green), *∆A0254_A* (light blue), *∆A0556* (light green). The wildtype is indicated by black arrows. Four mutants that are severely inhibited by 0.03 mg/ml bile acids are highlighted with a red bar.


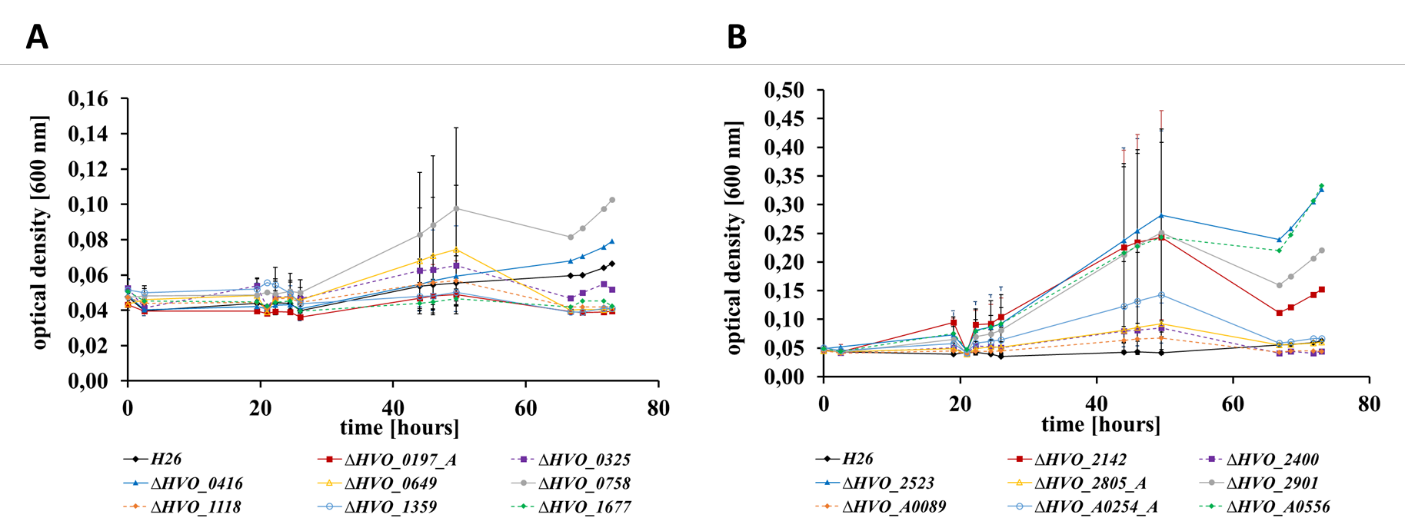


**Supplementary Figure S4.** Growth curves of the parent strain *H26* (black) and 16 deletion mutants (in color) exposed to bile acid [0.035 mg/ml]. **A.** Parent strain H26 (black) and deletion mutants *∆0197_A* (red), *∆0325* (purple), *∆0416* (dark blue), *∆0649* (yellow), *∆0758* (grey), *∆1118* (dark green), *∆1359* (light blue), *∆1677* (light green). **B.** Parent strain H26 and deletion mutants *∆2142* (red), *∆2400* (purple), *∆2523* (dark blue), *∆2805_A* (yellow), *∆2901* (grey), *∆A0089* (dark green), *∆A0254_A* (light blue), *∆A0556* (light green). Four mutants that grow in the presence of 0.035 mg/ml bile acids are highlighted by a red bar.
